# Four‐year follow‐up of weight loss maintenance using electronic medical record data: The PROPEL trial

**DOI:** 10.1002/osp4.70017

**Published:** 2024-10-19

**Authors:** Peter T. Katzmarzyk, Emily F. Mire, Ronald Horswell, San T. Chu, Dachuan Zhang, Corby K. Martin, Robert L. Newton, John W. Apolzan, Eboni G. Price‐Haywood, Dan Fort, Thomas W. Carton, Kara D. Denstel, the PROPEL Research Group

**Affiliations:** ^1^ Pennington Biomedical Research Center Baton Rouge Louisiana USA; ^2^ Ochsner Xavier Institute for Health Equity and Research New Orleans Louisiana USA; ^3^ Division of Academics Ochsner Center for Outcomes Research Ochsner Health New Orleans Louisiana USA; ^4^ Louisiana Public Health Institute New Orleans Louisiana USA

**Keywords:** diet, EMR, lifestyle behaviors, obesity, physical activity

## Abstract

**Rationale:**

Short‐term weight loss is possible in a variety of settings. However, long‐term, free‐living weight loss maintenance following structured weight loss interventions remains elusive.

**Objective:**

The purpose was to study body weight trajectories over 2 years of intensive lifestyle intervention (ILI) and up to 4 years of follow‐up versus usual care (UC).

**Methods:**

Data were obtained from electronic medical records (EMRs) from participating clinics. Baseline (Day 0) was established as the EMR data point closest but prior to the baseline date of the trial. The sample included 111 ILI and 196 UC patients. The primary statistical analysis focused on differentiating weight loss trajectories between ILI and UC.

**Results:**

The ILI group experienced significantly greater weight loss compared with the UC group from Day 100 to Day 700, beyond which there were no significant differences. Intensive lifestyle intervention patients who maintained ≥5% and ≥10% weight loss at 24 months demonstrated significantly greater weight loss (*p* < 0.001) across the active intervention and follow‐up.

**Conclusions:**

Following 24 months of active intervention, patients with ILI regained weight toward their baseline to the point where ILI versus UC differences were no longer statistically or clinically significant. However, patients in the ILI who experienced ≥5% or ≥10% weight loss at the cessation of the active intervention maintained greater weight loss at the end of the follow‐up phase.

**Clinical Trial Registration:**

ClinicalTrials.gov: NCT02561221.

## Graphical abstract

Following 24 months of active intervention, patients in an intensive lifestyle intervention regained weight toward their baseline to the point where differences between the intervention and usual care groups were no longer statistically or clinically significant during the 4‐year follow‐up phase. Patients in the intervention who experienced ≥5% or ≥10% weight loss at the end of the active intervention maintained greater weight loss at the end of the 4‐year follow‐up phase.

## 1 INTRODUCTION

Attaining meaningful weight loss in primary care settings has traditionally been challenging, and has resulted in limited success.^1^ Low‐ or moderate‐intensity counseling by primary care practitioners shows approximately 1–2 kg of weight loss after 12 months compared to usual care (UC). This small amount of weight loss is partly attributed to the use of low‐intensity, less effective interventions in primary care due to lack of provider training and limited resources (including time).^2^ However, higher‐intensity interventions delivered by trained professionals result in greater weight loss.^2^, ^3^, ^4^ Indeed, recent interventions that have deployed intensive behavioral interventions within primary care clinics have produced meaningful weight loss.^5^, ^6^, ^7^ For example, the Promoting Successful Weight Loss in Primary Care in Louisiana (PROPEL) trial produced 4.99% weight loss in the intensive lifestyle intervention (ILI) group compared to 0.48% weight loss in the UC group at 24 months^6^ While the weight loss obtained in PROPEL remained clinically significant at 24 months, the maximal weight loss was attained in the ILI at 6 months (7.3%), and patients regained 32% of their initial weight loss by month 24, despite continued intervention.^6^

Short‐term (i.e., 6 months) or even long‐term (i.e., 24 months) weight loss is possible in a variety of settings; however, long‐term free‐living weight loss maintenance following structured weight loss interventions remains elusive.^8^, ^9^ There is a need for studies that evaluate long‐term weight loss maintenance in real‐world settings after interventions have been deployed and discontinued and patients are left on their own to manage their own body weight based on what they have learned during an intervention.

One cost‐effective method for tracking body weight over time among patients is via electronic medical records (EMRs). Previous studies have shown good agreement between standardized measures of body weight and EMR‐obtained body weight.^10^, ^11^, ^12^, ^13^ For example, analyses of data from the PROPEL trial demonstrated a correlation of 0.988 between researcher‐measured and EMR‐obtained body weights, with a difference (EMR‐obtained minus researcher) of 0.63 (2.65 SD) kg.^13^ Thus, the use of EMR data may be a feasible approach to tracking weight loss maintenance in studies where the active intervention has ended.

The primary aim of this study was to examine changes in body weight over 4 years post‐intervention among PROPEL trial patients (6 years of total follow‐up from baseline) using data from EMRs. It is hypothesized that patients in the ILI will continue to slowly regain weight toward their baseline following the active intervention phase, and differences between the ILI and UC groups will no longer be clinically or statistically significant. The secondary aim was to study whether those in the ILI who maintained significant weight loss at the end of the active trial phase (i.e., ≥5% and ≥10% weight loss) maintained greater weight loss at the end of the follow‐up phase compared to those who experienced less weight loss during the active phase. It is hypothesized that those who lost more weight during the ILI will demonstrate greater weight loss maintenance during the observational follow‐up phase.

## 2 METHODS

PROPEL was conducted in 18 primary care clinics in Louisiana that served primarily low‐income patients.^6^ Clinics were randomly assigned to an ILI group or a UC group. Primary inclusion criteria included being 20–75 years of age, having a BMI between 30 and 50 kg/m^2^, and being affiliated with a participating clinic. Exclusion criteria included using weight‐loss medications, having a history of bariatric surgery, currently participating in a weight‐loss program, or experiencing a recent weight loss. Full eligibility criteria have been previously published.^14^

**FIGURE 1:**
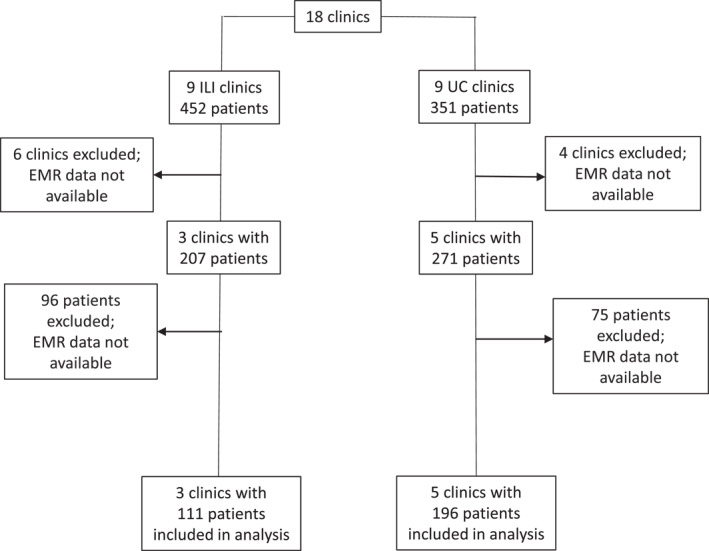
Flow diagram depicting the derivation of the analytic sample from the original PROPEL trial sample.

Figure 1 provides a description of the derivation of the sample for the current analysis. The full sample of the original PROPEL trial included 803 patients. Of these, 325 were patients at primary care clinics (n = 10) that did not contribute EMR data to this study. Of the remaining 478 patients, 346 individuals consented to allow access to their EMR data for follow‐up analyses. The final analytical dataset was further limited to patients who had a baseline measurement (an EMR value for weight within 90 days of baseline) plus at least 1 additional body weight recorded over a maximum follow‐up of 6 years (2 years active trial phase followed by 4 years post‐active trial phase), resulting in a sample size of 307 patients (111 ILI, 196 UC) available for analysis. There were 3666 body weight datapoints within the active trial phase (mean = 11.9 per patient), with an additional 5844 data points in the follow‐up phase (mean = 21.8 per patient). A total of 268 patients underwent an EMR visit with an associated body weight during the post‐active follow‐up phase. The sample that contributed EMR data was significantly older, had a lower proportion of females, had higher levels of income, and had a higher proportion of patients with Type 2 diabetes, compared to those with no EMR data. No differences between the two groups in baseline weight or BMI were observed (Table S1). In the original trial, only 4 patients (2 in ILI, two in UC) underwent bariatric surgery over the course of the study period.^6^ The protocol was approved by the Pennington Biomedical Research Center Institutional Review Board, and all patients provided written informed consent, including consent to access their EMR data during follow‐up.

### 2.1 Study arms

Patients in the ILI group received a comprehensive high‐intensity lifestyle intervention, consistent with the 2013 AHA/ACC/TOS Guidelines for Managing Overweight and Obesity in Adults.^15^ The PROPEL ILI was based on successful weight loss regimens such as the Diabetes Prevention Program,^16^ Look AHEAD,^17^ and CALERIE.^18^ Locally trained health coaches delivered the ILI in primary care clinics, with 24 weekly sessions in the first 6 months followed by monthly sessions for the remainder of the trial. During the initial 6 months, the objective was for patients to lose 10% of their initial body weight, followed by weight loss maintenance for the remaining 18 months. Patients and health coaches worked on goal setting and developing and adhering to customized diet and physical activity action plans.^14^

Patients in the UC group received health care through their primary care team. Patients in the UC group were also provided with six newsletters (three per year) on selected topics related to health, which also included local community health promotion events and resources.

### 2.2 Outcomes

The primary outcome of interest was percent weight loss from baseline. Data were extracted from the EMR of the health care providers, and data were harmonized to a common format. The baseline EMR visit for each patient was established as the EMR visit closest but prior to their baseline date in PROPEL (within the prior 90 days). For the analyses comparing weight loss outcomes among those who lost ≥5% and ≥10% body weight versus lower amounts of weight loss, we relied on our technician‐assessed body weights at baseline and 24 months to categorize the patients.

### 2.3 Covariates

Age (years) was computed from birth and observation dates. Sex assigned at birth (female/male) and race (Black, Other) were obtained from self‐reporting questionnaire at baseline.

### 2.4 Statistical analysis

The primary statistical analysis focused on differentiating weight loss trajectories between the ILI and UC groups. To capture non‐linear relationships in the data, we employed cubic spline regression analysis with a B‐spline basis for examining long‐term weight changes over ∼6 years post‐baseline (∼4 years post‐intervention) in PROPEL trial patients. The time effect of days between each EMR measurement and EMR baseline was constructed into the spline effect. The placement of three knots (days 547, 1094, and 1641) was based on quartiles from day 0 (baseline) to the last day 2186 to capture inflection points systematically. Additionally, the analysis accounted for within‐subject correlation and included a random cluster effect of the clinic. The statistical model was formulated as follows:

$${\%\text{WL}}_{ijtn}=\beta_{0}+\beta_{1}{\text{Group}}_{i}+f\left(t\right)\times {\text{Group}}_{i}+b_{j}+\epsilon_{ijtn}$$

where %WL_*ijtn*_ represented the percent weight loss of the *n*th individual of clinic *j* in group *i* at time *t*, *b* was the random cluster term, and the spline functions $f\left(t\right)=\sum_{k=1}^{3}\beta_{2k}B_{k}\left(t\right)$ where *B*_*k*_(*t*) was the *k*th B‐spline basis.

We employed a SIMULATE adjustment for multiple comparisons at each 100‐day interval between the ILI and UC groups. Furthermore, a step‐down adjustment was implemented for pairwise comparisons, enhancing the power of multiple comparisons by considering logical constraints among hypotheses and correlations among test statistics. SAS software, version 9.4 (SAS Institute) was used to conduct all data analyses.

Weight loss over the follow‐up period was compared between those that lost ≥5% body weight versus those that did not, and between those that lost ≥10% body weight versus those that did not. The analytical strategy was the same as that used for the primary aim of comparing weight loss between the ILI and UC patients described above. In addition, we used the same approach to test for differences by sex (men vs. women), race (Black vs. Other) and age (<52years vs. ≥ 52 years).

## 3 RESULTS

Descriptive baseline characteristics of the sample are presented in Table 1. No appreciable differences between the ILI and UC groups were observed, with the exception that a higher proportion of diabetes patients was seen in the UC group (35.7%) compared to the ILI group (22.5%).

**TABLE 1:** Descriptive characteristics of the sample at baseline.

	UC	ILI	Total
Patients, *n*	196	111	307
Age, y	54.1 (12.1)	53.9 (11.4)	54.1 (11.8)
Weight, kg	102.7 (18.0)	103.6 (18.6)	103.0 (18.2)
BMI, kg/m^2^	36.9 (4.7)	37.3 (4.8)	37.1 (4.7)
Female sex, *n* (%)	143 (73.0)	89 (80.2)	232 (75.6)
Race, *n* (%)			
Black	123 (62.8)	80 (72.1)	203 (66.1)
White	62 (31.6)	26 (23.4)	88 (28.7)
Other	11 (5.6)	5 (4.5)	16 (5.2)
Hispanic, *n* (%)	11 (5.6)	4 (3.6)	15 (4.9)
Income, *n* (%)			
<$10,000	31 (16.2)	10 (9.2)	41 (13.4)
$10,000–19,999	34 (17.8)	16 (14.7)	50 (16.3)
$20,000–39,999	43 (22.5)	29 (26.6)	72 (23.5)
$40,000–59,999	29 (15.2)	18 (16.5)	47 (15.3)
≥$60,000	54 (28.3)	36 (33.0)	90 (29.3)
Missing	5 (2.6)	2 (1.8)	7 (2.3)
Health literacy, *n* (%)			
≤8th grade	63 (32.1)	32 (28.8)	95 (30.9)
≥9th grade	133 (67.9)	79 (71.2)	212 (69.1)
Food insecurity, *n* (%)	68 (34.7)	30 (27.0)	98 (31.9)
Diabetes, *n* (%)	70 (35.7)	25 (22.5)	95 (30.9)

*Note*: Values are reported as means (SD) unless otherwise indicated.

**FIGURE 2:**
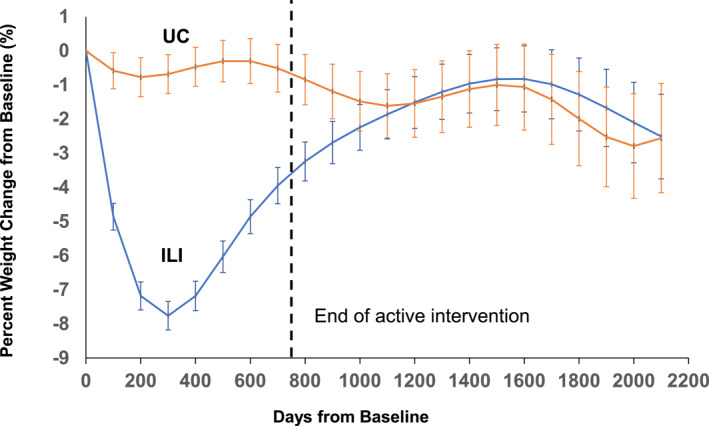
Percent weight loss from baseline in the Intensive Lifestyle Intervention and Usual Care groups. Error bars represent standard errors. The vertical dotted line indicates the end of the active study phase; *p < 0.05 between groups.

The primary analysis revealed statistically significant differences between ILI and UC groups at specific time points. Figure 2 provides a graphical representation of the percent changes in weight from baseline in the two groups. The ILI group demonstrated significantly more weight loss compared to the UC group during the period from Day 100 to Day 700, which corresponds to the active intervention phase (Table 2). The largest difference in weight loss between the ILI and UC groups was observed on day 300 (−7.08 ± 0.71%, *p* < 0.001). Differences between the ILI and UC groups were not statistically significant beyond Day 700, suggesting a trend of weight regain in the ILI group during this post‐intervention period. Figure S1 visually depicts the non‐linear patterns of body weight trajectories over time for both groups, taking into account the non‐independence of multiple data points per subject. The observed curvature supported the statistical findings of significant group differences from day 100 to day 700 with the maximum difference highlighted on day 300.

**TABLE 2:** Estimated weight change (%) by group and group difference at each 100‐day time point.

Time	ILI WL% ± SE	UC WL% ± SE	Diff ± SE (95% adjusted CI)	Adjusted *p*
Day 100	−4.86 ± 0.53	−0.58 ± 0.39	−4.27 ± 0.66 (−6.37, −2.17)	<0.001
Day 200	−7.18 ± 0.57	−0.77 ± 0.41	−6.41 ± 0.70 (−8.63, −4.19)	<0.001
Day 300	−7.76 ± 0.57	−0.68 ± 0.42	−7.08 ± 0.71 (−9.33, −4.83)	<0.001
Day 400	−7.18 ± 0.57	−0.47 ± 0.43	−6.71 ± 0.72 (−8.98, −4.44)	<0.001
Day 500	−6.03 ± 0.61	−0.30 ± 0.46	−5.73 ± 0.76 (−8.15, −3.30)	<0.001
Day 600	−4.86 ± 0.66	−0.30 ± 0.50	−4.56 ± 0.83 (−7.20, −1.92)	0.001
Day 700	−3.95 ± 0.70	−0.51 ± 0.53	−3.44 ± 0.88 (−6.24, −0.63)	0.010
Day 800	−3.24 ± 0.74	−0.84 ± 0.57	−2.40 ± 0.94 (−5.38, 0.58)	0.082
Day 900	−2.69 ± 0.80	−1.19 ± 0.62	−1.50 ± 1.01 (−4.71, 1.71)	0.385
Day 1000	−2.24 ± 0.87	−1.48 ± 0.67	−0.77 ± 1.10 (−4.25, 2.71)	0.837
Day 1100	−1.86 ± 0.94	−1.61 ± 0.72	−0.25 ± 1.18 (−4.00, 3.50)	0.985
Day 1200	−1.51 ± 0.99	−1.54 ± 0.76	0.03 ± 1.25 (−3.94, 4.01)	0.999
Day 1300	−1.20 ± 1.05	−1.34 ± 0.81	0.14 ± 1.33 (−4.06, 4.35)	0.994
Day 1400	−0.96 ± 1.12	−1.12 ± 0.86	0.16 ± 1.41 (−4.32, 4.64)	0.993
Day 1500	−0.83 ± 1.19	−1.00 ± 0.92	0.17 ± 1.50 (−4.60, 4.94)	0.994
Day 1600	−0.82 ± 1.26	−1.06 ± 0.97	0.24 ± 1.59 (−4.80, 5.28)	0.988
Day 1700	−0.98 ± 1.32	−1.42 ± 1.01	0.44 ± 1.66 (−4.83, 5.71)	0.985
Day 1800	−1.28 ± 1.38	−1.99 ± 1.07	0.71 ± 1.75 (−4.84, 6.25)	0.965
Day 1900	−1.67 ± 1.46	−2.52 ± 1.13	0.85 ± 1.85 (−5.00, 6.71)	0.945
Day 2000	−2.10 ± 1.53	−2.79 ± 1.18	0.70 ± 1.93 (−5.43, 6.82)	0.976
Day 2100	−2.51 ± 1.60	−2.56 ± 1.24	0.05 ± 2.02 (−6.38, 6.47)	0.999

**FIGURE 3:**
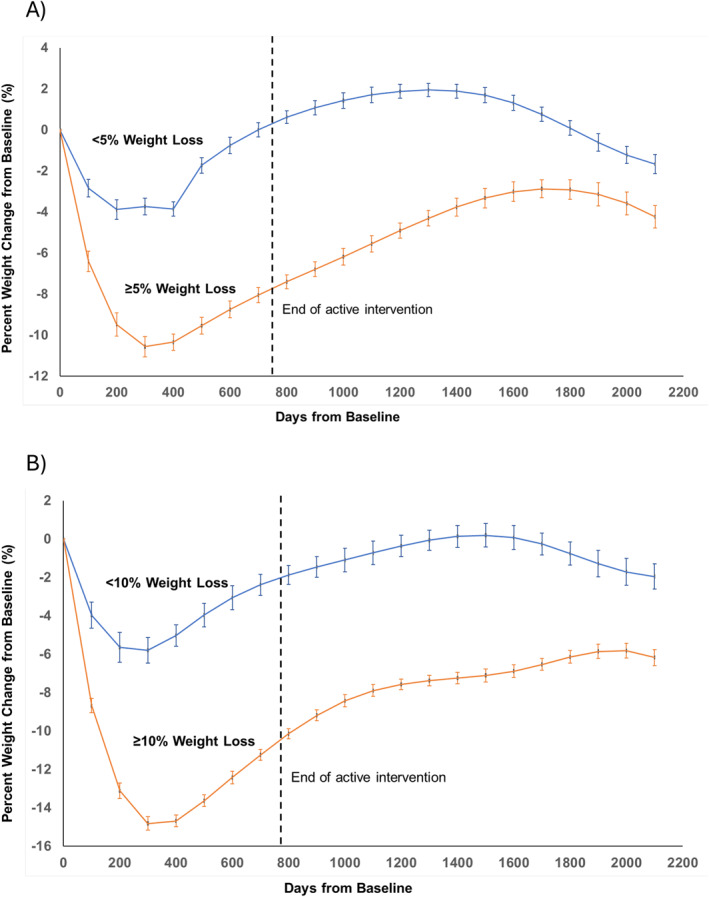
Percentage weight loss in the Intensive Lifestyle Intervention group among those who lost (A) ≥5% body weight and (B) ≥10% body weight between baseline and 12 months versus those who did not. Error bars represent standard errors. The vertical dotted line indicates the end of the active study phase; *p < 0.05 between groups.

A total of 69 (62%) of the ILI patients experienced ≥5% weight loss, and 31 (28%) of the ILI patients maintained ≥10% weight loss at 24 months Figure 3 presents the weight loss during follow‐up among ILI patients who experienced ≥5% and ≥10% weight loss over 24 months in the intervention versus those that did not, respectively. Patients in the group who experienced ≥5% weight loss at 24 months demonstrated significantly greater weight loss (*p* < 0.001) across the active intervention and follow‐up phases compared with those who experienced less weight loss. Likewise, patients in the group who experienced ≥10% weight loss at 24 months demonstrated significantly greater weight loss (*p* < 0.0001) across the active intervention and follow‐up phases compared with those who experienced less weight loss. At the end of follow‐up (2100 days or ∼5.8 years), those who experienced ≥5% weight loss maintained 2.56 (95% CI: 0.47–4.65) kg more weight loss compared to those who lost less weight, and those who experienced ≥10% weight loss maintained 4.22 (95% CI: 1.94–6.50) kg more weight loss compared to those who lost less weight.

Figures S2–S4 present weight loss stratified by sex, race and age. In general, women tended to lose more weight than men throughout the study, but there were only five timepoints when these differences reached statistical significance. There were no differences in weight loss among younger and older adults throughout the active intervention phase, but older adults tended to lose some weight near the end of the follow‐up phase. Weight loss was quite similar among Black and Other races during the first 400 days of the intervention, after which Black patients regained more weight at day 1400.

## 4 DISCUSSION

The main results indicate that the observed differences in weight loss between the ILI and UC groups in the PROPEL trial largely disappeared after the suspension of the intervention, but patients in the ILI group who lost more weight (i.e., ≥5% and ≥10%) experienced greater longer‐term weight loss maintenance than patients who experienced less weight loss during the active intervention.

The study results, which show significant weight regain following the suspension of an ILI, are largely consistent with the existing literature. For example, a meta‐analysis of 29 studies with an initial intervention period of 19 weeks (range of 8–30 weeks) indicated that patients regained approximately 56% of their initial weight loss at two years, and 79% of their weight loss at five years.^8^ Similarly, a meta‐analysis of 12 studies showed that 1 year following an intervention, approximately 46% of the initial weight loss was regained.^9^ Overall, these observational results indicate that weight regain occurs in free‐living conditions following an active intervention. However, the results from weight loss maintenance trials designed to minimize weight regain indicate that behavioral interventions targeting lifestyle changes show small but significant beneficial effects.^19^

A prior meta‐analysis of 12 studies also reported significantly greater net weight loss after follow‐up among intervention groups that achieved >10% weight loss.^9^ These results corroborate those that were obtained in the present study, where patients that initially lost ≥5% and ≥10% body weight maintained greater weight loss throughout the follow‐up period compared to patients who lost less weight. These results mirror the association between short‐term weight loss and subsequent weight change during active interventions. For example, a previous analysis from PROPEL demonstrated that greater weight loss in the initial two to 8 weeks of the intervention was positively associated with 24 months weight loss (*p* < 0.01).^20^ Taken together, these results suggest that longer‐term weight loss or maintenance, either during or after an active intervention, are predicated to some extent on prior weight loss success.^21^

The PROPEL ILI was adapted from the DPP and Look AHEAD ILIs. The DPP and Look AHEAD interventions were delivered largely through academic health centers rather than primary health care settings per se. In 2006, the National Heart, Lung, and Blood Institute funded three pragmatic trials of behavioral interventions for weight loss that were conducted in primary care settings.^22^ The results of these trials demonstrated the viability of primary care as a potential setting for weight loss, and that the results differed according to intensity and mode of intervention.^4^ Results from the 15‐year DPP Outcomes Study (DPPOS) indicated that mean weight in the ILI increased for about 2 years following the cessation of the active intervention, and plateaued at approximately 2–3 kg of weight loss from baseline for several years.^23^ Results from the Look AHEAD post‐intervention observational follow‐up study (∼8 years from cessation of active intervention) indicated that ILI patients continued to lose weight (−3.0% from end of active intervention).^24^ While DPP, Look AHEAD and PROPEL shared many intervention features in common, there are important differences during the post‐intervention follow‐up period that may account for differential weight regain experiences among patients in these trials. For example, during the DPPOS, group lifestyle sessions were offered quarterly to all participants, and ILI patients were also offered additional group programs which reinforced behavioral self‐management activities twice per year.^23^ Further, both DPPOS and the Look AHEAD follow‐up study involved in‐person clinical assessment of patients, while the PROPEL follow‐up was conducted using data from EMRs and was purely a naturalistic observational follow‐up. The degree to which continued contact with the research team over the follow‐up period in DPPOS and Look AHEAD influenced the results is unknown.

The mechanisms underlying weight regain following successful weight loss remain elusive; however, the combination of living in an obesogenic environment and metabolic adaptations that drive body regulation are central to the phenomenon.^25^ Metabolic adaptations that accompany weight loss and promote subsequent weight regain occur on both the energy expenditure and energy intake sides of the energy balance equation. These adaptations result from central hedonic and homeostatic mechanisms in the brain interfacing with the gut and other peripheral tissues.^26^ There is a critical need for further research to better understand the pathophysiology of weight regain within the context of weight loss maintenance over the long term.^25^

Several strengths and limitations of this study exist. A key strength is the reliance on real‐world EMR data to monitor the trends in body weight over 6 years. This approach minimizes concerns about recall bias among patients who may have trouble accurately reporting changes in their body weight over long time periods. On the other hand, the EMR dataset is somewhat limited in that “carry‐forward” weight from one clinical visit to the next may have impacted the analysis. The degree to which carryforward weights may have influenced the results is not known; however, a prior analysis of data from the PROPEL trial reported a very high correlation between researcher‐measured and EMR‐obtained body weights.^13^ Further, the differences in EMR‐based weight loss between ILI and UC during the active phase of the trial largely mirror those from the primary PROPEL analysis, which utilized research technician‐assessed body weights.^6^ Another limitation is that we did not have specific time points where EMR data could be aligned (i.e., 6, 12, 24 months); therefore, the body weight estimates were derived from statistical models which may have been influenced by model selection. For a variety of reasons, EMR data were available for only 8 of the original 18 PROPEL clinics (and 38% of original PROPEL patients). The degree to which this biases the results of the analyses is not known; however, the results for the first 24 months of active intervention presented here mirror those from the larger sample.^6^, ^13^ Finally, the results of this study were derived from primary care settings, and the generalizability of the results to other populations or other methods of weight loss such as pharmacotherapy or bariatric surgery are not known.

In summary, the results of this study indicate that weight regain occurred after the suspension of an active ILI; however, patients who lost more weight during the active intervention demonstrated greater weight loss maintenance compared with patients who achieved less weight loss during the active intervention. The results indicate that long‐term weight loss is difficult for many patients, and further research is required to develop intervention programs to support their initial weight loss efforts in the future. These results also highlight the importance of achieving early weight loss success, and support the recommendation of the 2013 AHA/ACC/TOS Obesity Guidelines to utilize “high‐intensity” programs as first‐line therapy for obesity treatment and management.^15^

## AUTHOR CONTRIBUTIONS

**Peter T. Katzmarzyk:** Conceptualization, funding acquisition, investigation, writing—original draft, supervision. **John W. Apolzan:** Funding acquisition, investigation, writing—review & editing. **Thomas W. Carton:** Data curation, writing—review & editing. **San T. Chu,** Data curation, writing—review & editing. **Kara D. Denstel:** Investigation, data curation, writing—review & editing, project administration. **Dan Fort:** Data curation, writing ‐ review & editing. **Ronald Horswell:** Data curation, writing—review & editing. **Corby K. Martin:** Funding acquisition, investigation, writing—review & editing. **Emily F. Mire:** Formal analysis, investigation, data curation, writing—review & editing. **Robert L. Newton:** Funding acquisition, investigation, writing—review & editing. **Eboni G. Price‐Haywood:** Writing—review & editing, supervision. **Dachuan Zhang:** Data analysis, data interpretation. All authors have final approval of the submitted and published versions.

## CONFLICT OF INTEREST STATEMENT

PK, RN, CM, RH and DF report NIH grants to their institution. PK, CM and JA report research contracts between WW International and their institution. JA and CM report research grants from USDA, NSF, Lilly, and FFAR, and their institution. CM reports a research grant from Kroger Co. Zero Hunger/Zero Waste Foundation and his institution. JA reports a research grant between the HASS Avocado Board and his institution. DF reports PCORI contracts and CDC grants to his institution. TC reports a research award from PCORI. CM reports speaking honoraria and travel support from Indiana University, UAB, Brigham Young University, University of Kansas Medical Center, and University of Southern California JA reports a speaking honorarium and travel support from WW International/Abbott. DF reports a speaking honorarium from Tulane University. CM reports royalties from ABGIL, consulting fees from Wondr Health, EHE Health, UAB, the Commission on Dietetic Registration, and University of Nebraska, and participation on the DSMB of a weight loss trial at Duke University. No other conflicts of interest were reported.

## Supporting information

Supporting Information S1

## Data Availability

The protocol and a deidentified dataset used in this study are available from the corresponding author upon reasonable request. Proposals should be directed to peter.katzmarzyk@pbrc.edu. A data use agreement with Pennington Biomedical Research Center is required.
