# Supplementary material for: Four‐year follow‐up of weight loss maintenance using electronic medical record data: The PROPEL trial
Source: Obes Sci Pract. 2024 Oct 19;10(5):e70017. doi: 10.1002/osp4.70017 (PMC11490219; doi:10.1002/osp4.70017)
Supplement: Supplementary file 1 — Supporting Information S1 [file OSP4-10-e70017-s001.pdf]

Four-Year Follow-up of Weight Loss Maintenance Using Electronic Medical Record Data: The PROPEL Trial

Peter T. Katzmarzyk, PhD[1], Emily F. Mire, MS[1], Ronald Horswell, PhD[1], San T. Chu, MS[1], Dachuan Zhang, MS[1], Corby K. Martin, PhD[1], Robert L. Newton, Jr., PhD[1], John W. Apolzan, PhD[1], Eboni G. Price-Haywood, MD, MPH[2,3], Dan Fort, PhD[3], Thomas W. Carton, PhD[4], and Kara D. Denstel, PhD, MPH[1] for the PROPEL Research Group

[1]Pennington Biomedical Research Center, Baton Rouge, LA
[2]Ochsner Xavier Institute for Health Equity and Research, New Orleans, LA
[3]Ochsner Center for Outcomes Research, Division of Academics, Ochsner Health, New Orleans, LA
[4]Louisiana Public Health Institute, New Orleans, LA

Table S1. Comparison of baseline descriptive characteristics among PROPEL patients with and without EMR follow-up data.

	Patients with EMR Follow-Up	Patients without EMR Follow-Up	P for Difference*
**Patients, n**	307	496	
**Age, y**	54.1 (11.8)	46.5 (13.0)	<0.0001
**Weight, kg**	103.0 (18.2)	101.5 (15.6)	0.21
**BMI, kg/m^2**	37.1 (4.7)	37.3 (4.6)	0.45
**Female Sex, n (%)**	232 (75.6)	446 (89.9)	<0.0001
**Race, n (%)**			0.59
**Black**	203 (66.1)	337 (67.9)	
**White**	88 (28.7)	120 (24.2)	
**Other**	16 (5.2)	39 (7.9)	
**Hispanic, n (%)**	15 (4.9)	17 (3.4)	0.30
**Income, n (%)**			<0.0001
**<$10,000**	41 (13.4)	115 (23.2)	
**$10-19,999**	50 (16.3)	118 (23.8)	
**$20-39,999**	72 (23.5)	119 (24.0)	
**$40-59,999**	47 (15.3)	70 (14.1)	
**≥$60,000**	90 (29.3)	64 (12.9)	
**Missing**	7 (2.3)	10 (2.0)	
**Health Literacy, n (%)**			0.93
**≤8th grade**	95 (30.9)	152 (30.7)	
**≥9th grade**	212 (69.1)	344 (69.4)	
**Food Insecurity, n (%)**	98 (31.9)	149 (30.0)	0.57
**Diabetes, n (%)**	95 (30.9)	112 (22.5)	0.009

*Significant differences were determined with a two-samples t-test for continuous variables and a chi-square test for categorical variables.

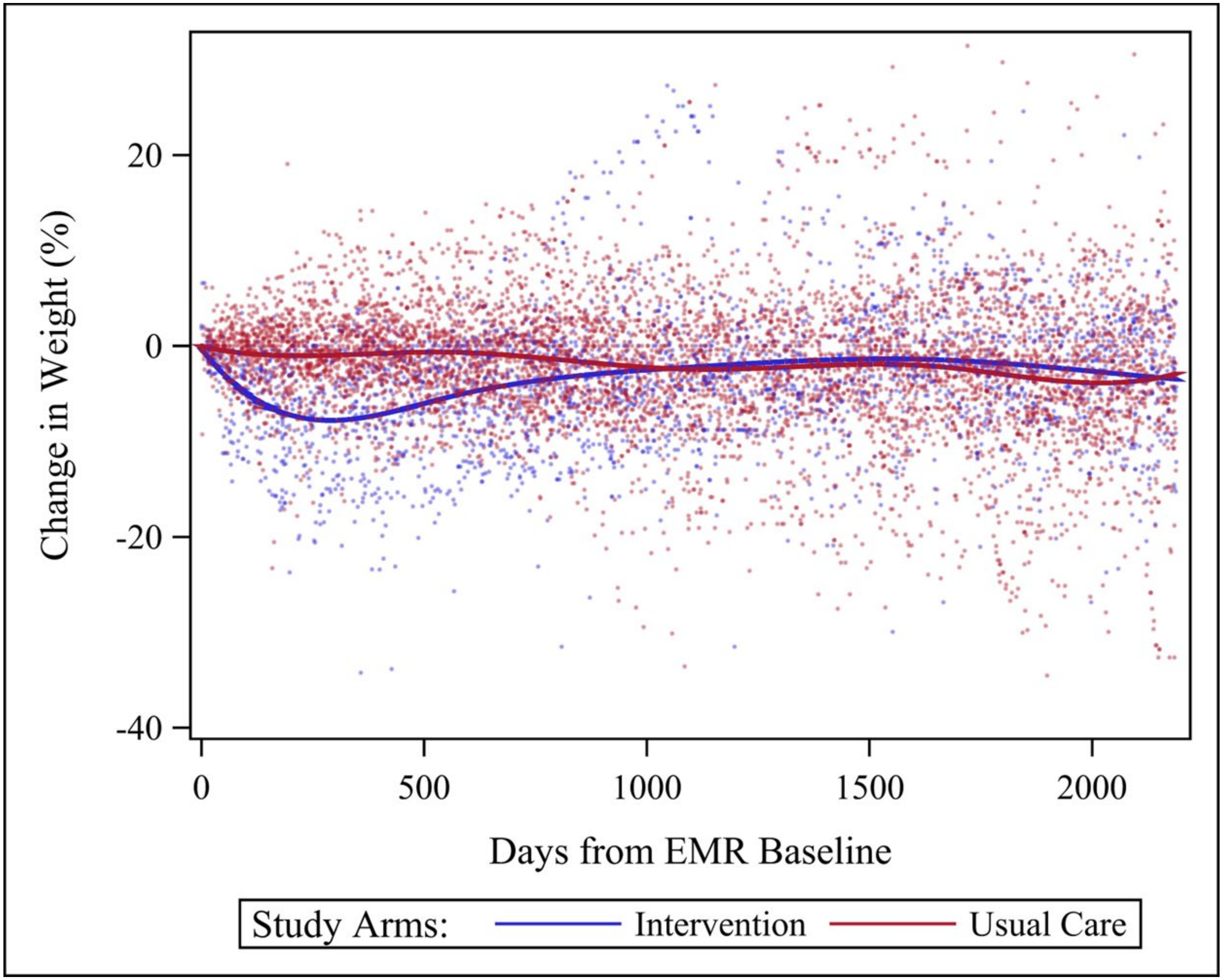

Figure S1. Individual data points and trends over time in the Intensive Lifestyle Intervention and Usual Care groups. Data obtained from electronic medical records (EMRs).

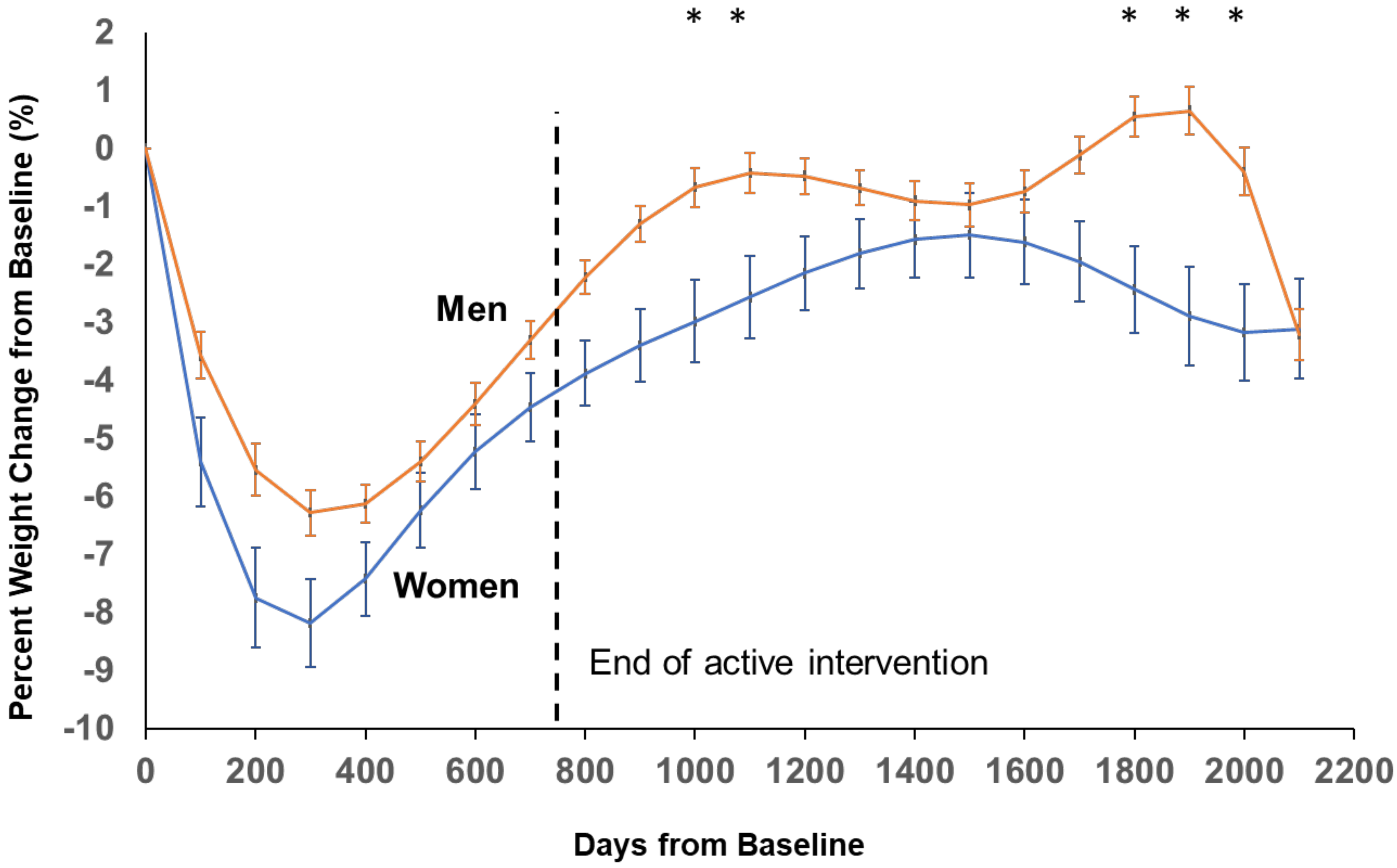

Figure S2. Percent weight loss from baseline in men and women. Error bars represent standard errors. The vertical dotted line indicates the end of the active study phase; *P<0.05 between groups.

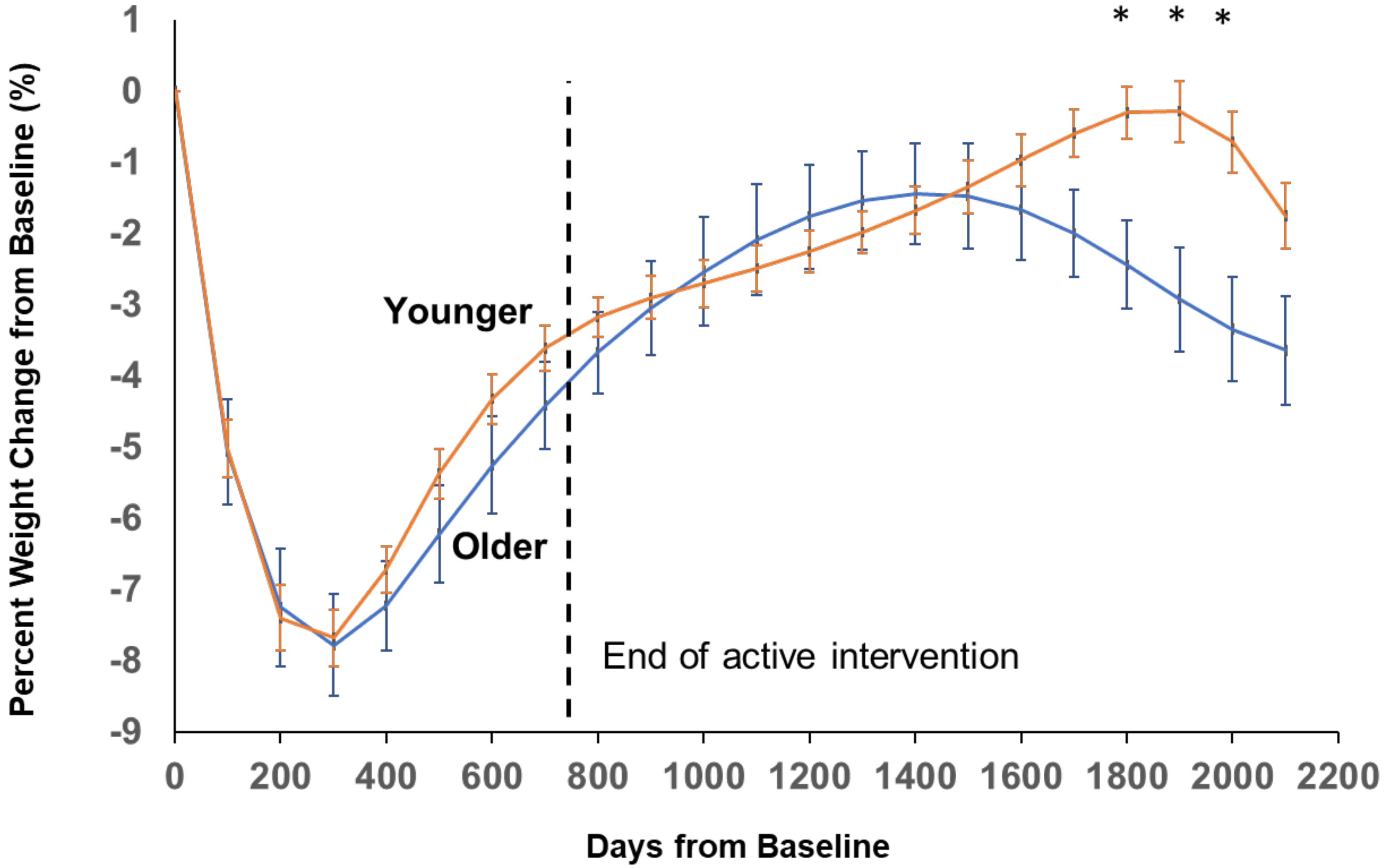

Figure S3. Percent weight loss from baseline in younger (<52 y) and older (≥52 y) age groups. Error bars represent standard errors. The vertical dotted line indicates the end of the active study phase; *P<0.05 between groups.

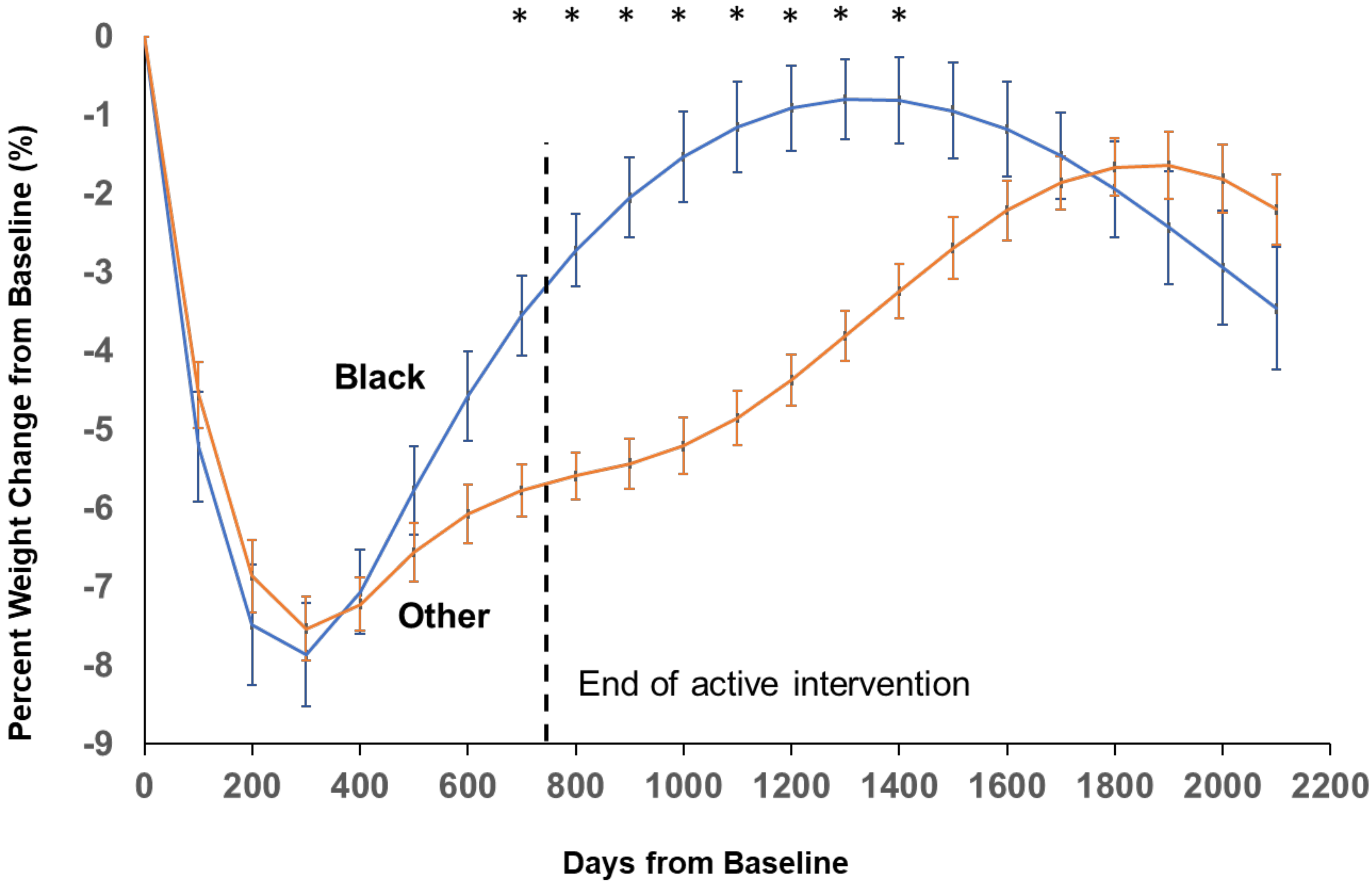

Figure S4. Percent weight loss from baseline in Black and Other races. Error bars represent standard errors. The vertical dotted line indicates the end of the active study phase; *P<0.05 between groups.
